# Complexity, Natural Selection and the Evolution of Life and Humans

**DOI:** 10.1007/s10699-014-9358-y

**Published:** 2014-05-03

**Authors:** Börje Ekstig

**Affiliations:** Department of Education, Uppsala University, Uppsala, Sweden

**Keywords:** Complexity, Natural selection, Evolution, The Tree of Life, Human species, Language

## Abstract

In this paper, I discuss the concept of complexity. I show that the principle of natural selection as acting on complexity gives a solution to the problem of reconciling the seemingly contradictory notion of generally increasing complexity and the observation that most species don’t follow such a trend. I suggest the process of evolution to be illustrated by means of a schematic diagram of complexity versus time, interpreted as a form of the Tree of Life. The suggested model implies that complexity is cumulatively increasing, giving evolution a direction, an arrow of time, thus also implying that the latest emerging species will be the one with the highest level of complexity. Since the human species is the last species evolved in the evolutionary process seen at large, this means that we are the species with the highest complexity. The model implies that the human species constitutes an integral part of organic evolution, yet rendering us the exclusive status as the species of the highest complexity.

## Introduction

There is a widespread notion that life has increased its complexity throughout evolutionary history. However, there is a problem with all discussions of complexity in that there is no definition generally agreed on. Nonetheless, complexity has become a concept of fundamental importance for the development of contemporary science as Santos (2013) empathizes in his discussion of the philosophical aspects of the concept.

Regarding the lack of definition, science has actually always had to struggle with definitions. In many cases, progress has started with measurements. Thus in the seventieth century the concept of force wasn’t well understood. Then Newton performed a measurement of acceleration related to the movement of the moon. On account of this measurement he could define the concept of force thus opening for the rapid development of physics. Likewise, in the ninetieth century, the concept of energy was nebulous. In that situation James Prescott Joule performed a famous experiment in which he compared mechanical energy and heat and so he could lay down a unit for the measurement of heat, thus initiating the science of thermodynamics.

I would in this context like to mention that I have suggested a measurement of the relative values of complexity (Ekstig 2010a, 2011). It’s important to note that this measurement didn’t rely on or lead to a stringent definition. Anyhow, in the present analysis the results of these measurements are not used, nor are they necessary prerequisites. I merely base my analysis in the present paper on qualitative reasoning although the conclusions are quite compatible with my previous measurements.

Charles Lineweaver, Paul Davis and Michael Ruse have in their book *Complexity and the Arrow of Time* (Lineweaver et al. 2013) brought together a number of scientists unveiling their joint effort in exploring the concept of complexity. The contributors grapple with the notion of increasing complexity in the cosmological and biological spheres, and are frustrated by the lack of a definition of complexity generally agreed on. But, as the editors ask, even without a definition or a way to measure it, isn’t it qualitatively obvious that biological complexity has increased? Do we really need to wait for a precise definition to think about complexity and its limits? (p. 5).

The notion of increasing complexity is associated to the question of the direction of evolution, “the arrow of time”, a question that has been a challenging issue during all the history of evolutionary theory as comprehensively reviewed by Ruse (2013).

If natural selection has forced complexity to increase, as many authors seem to maintain, how then to explain the fact that the oldest organisms of today, i.e. those having been exposed to natural selection for the longest time, are the most primitive? Isn’t the opposite rather to be expected? Or, as Lamarck asked already when the very idea of evolution was quite new, if the active power of nature compels life to mount steadily up the chain of being, how can we still see the complete hierarchy today? Why haven’t all living things raised themselves to the same level as man? (Bowler 1989 p. 85). Although Lamarck’s views now are obsolete, not to say outright taboo, I find these questions still challenging. Similarly one may ask how a general trend of increasing complexity can be compatible with the observation that most species show only marginal changes during most of their evolutionary history.

## What is Complexity?

The biological world is bewilderingly complex. But what, really, is complexity? Many attempts at definitions are found in Mitchell’s (2009) comprehensive account. These attempts testify the difficulties in defining complexity in terms of more fundamental concepts. I therefore suggest that we at present have to be content with a description by means of common and easily understood concepts. As a very simple first attempt of an answer, I would like to use a formulation by McShea and Brandon (2010), saying that complexity means the number of parts or the amount of differentiation among parts within an individual.

Features of living organisms such as the locomotive organs, internal organs like the heart and the kidneys, the sense organs, the ability to react on external stimuli, the social behavior, the understanding of symbols, the use of language, and the intelligence are examples of features displaying complexity according to the suggested meaning. The natural variation of such capabilities in a population is what natural selection is acting on and it is reasonable to think that an enhanced capability of such features in many cases give the organism a reproductive advantage, thus implying that increased complexity is driven by natural selection.

Maybe an example may clarify the reasoning. Let us look at the evolution of the heart. In the primitive fish the chambers are arranged sequentially, later developing into an S-shape form in ray-finned fish. In reptiles such as crocodiles, this form was during several intermediate steps developed into a true four-chamber heart, making possible the separation of the de-oxygenated blood for the lungs and the oxygenated blood for to the rest of the body. This means an increase of the number of parts and of the amount of differentiation among these parts of the heart, in other words an increasing level of complexity. The changes are driven by their reproductive advantage, in this case even making possible the invasion of a new habitat, the transition to terrestrial life.

The contention of complexity as driven by natural selection is, I think, appropriate even in the many situations where the environmental contingencies offer no advantage of a change because, in such cases, natural selection is nonetheless active in eliminating unfavorable changes, thus keeping the organism alive at an unchanged level of complexity. I will come back to this issue.

My aim of the present paper is to investigate the evolutionary process in a broad sense and its coupling to the concept of complexity. Especially, I will show that the analysis of complexity in its connection to natural selection solves the enigma posed by Lamarck as discussed above. Finally, I extend the analysis to the human species, thus uniting the evolution of humanity with the rest of animal evolution.

## Some Views on Increasing Complexity

With his classical book *What is Life?* physicist Schrödinger (1955) was maybe the first to call attention to the problem of how the process of life can proceed in increasing its level of complexity, while obviously violating the second law of thermodynamics. The thermodynamic point of view is recently taken up with the idea of natural selection as forcing genomes to behave like a Maxwellian Demon, thus causing genomic complexity to increase (Adami et al. 2000). Still more provocatively, Wolpert (2013 p. 249) speculates if the second law can be an underlying driver of complexity rise in many biological systems. Might it somehow be, as he asks, that the second law, which increases disorder in a closed system, not only allows for open subsystems to increase their order, but also actually drives them to do so?

Paul Davies agrees to the notion of a generally increasing complexity in stating that the Tree of Life always “points upwards”. “If we take as a measure of complexity change, not successive instantaneous sums over all extant species, but the directionality of branching, then there is clearly, for unexceptional reasons, a growth of complexity with time” (Davies 2013 p. 30).

John Maynard Smith, and his co-author, Eörs Szathmáry, begin their book *The Major Transitions in Evolution *with the following statements: “Living organisms are highly complex /.../ This book is about how and why this complexity has increased in the course of evolution /.../Our thesis is that the increase has depended on a small number of major transitions /.../” (Maynard Smith and Szathmáry 1995 p. 3).

Some authors include cultural evolution in their analyses. Thus Maynard Smith and Szathmáry also include the evolution of human language as one of the major transitions. In discussing the applicability of complexity to human culture, Laszlo (2009), in his analysis of the general evolutionary system theory, postulates a universal flow towards ever-increasing complexity, affirming the rate of evolutionary change to be accelerating at the socio-cultural level largely because the motors of memetic change rely on information sharing (rather than energy/matter exchange) and therefore allows for faster rates of change than the biophysical level of genetic change.

It should also be mentioned that there are scientists rejecting the notion of increasing complexity. At least, that is how I interpret Robert Trivers’s statement: “There exists no objective basis on which to elevate one species above another. Chimp and human, lizard and fungus, we have all evolved over some three billion years by the process known as natural selection.” (Trivers 1976).

However, in the present article I challenge Trivers’s view in showing that it actually is natural selection that elevates species to successively higher levels.

In all his writing Richard Dawkins avoids talking about complexity. He fervently rejects the idea that contemporary animals could be ranked on a “higher or lower” scale (Dawkins 1992 p. 263) although he is somewhat more open to the concept of progress, especially as result of arms race (Dawkins 2004 pp. 496–497).

## The Debate of the Mechanism of Increasing Complexity

In contrast to Dawkins, Stephen Jay Gould comprehensibly discusses complexity and its increasing mode during life’s expansion. However, he explicitly denies increasing complexity to be driven by natural selection. In referring to a picture of the distribution of creatures as a skewed curve with simple organisms to the left and the most complex to the right he states that “the right tail is not a fundamental thrust produced by the superiority of complex forms under natural selection”. Instead he contends that “the most venerable evidence for general progress—the increasing complexity of the most complex—becomes a passive consequence of growth in a system with no directional bias whatever in the motion of its components” (Gould 1996 p. 171–173).

**Fig. 1:**
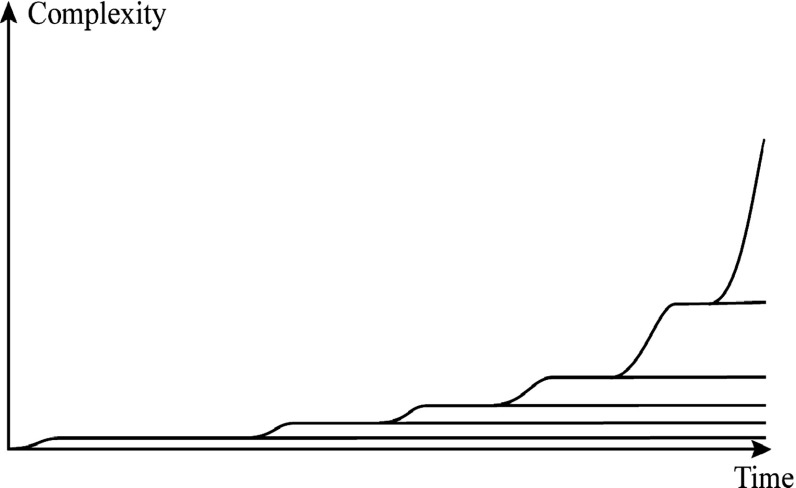
A principal diagram of complexity versus time. Rationales for its construction and interpretation are discussed in the text

For the explication of such a passively increasing complexity, Gould discusses a kind of a diffusion process which he explains by means of a metaphor known as the “drunkard’s walk” (ibid. pp. 149–150) that has turned out to have had great general impact. In this metaphor, a drunkard is staggering along the sidewalk at random. But on one side there is the wall of the bar making a limit that causes him to stray away from the wall. Gould means that in an analogous way, living organisms are drifting toward higher complexity at random, because there is a limit of minimal complexity. Therefore, Gould maintains, there is no need of a drive by natural selection. Applied to my model, this limit of minimal complexity is the time axis in the diagram of Fig. 1, corresponding to zero complexity. However, in Gould’s metaphor, nothing prevents the drunkard to pass over his own previous footprints over and over again, thus occasionally bringing him again close to the wall. This implies, in the evolutionary process, that a species of high complexity occasionally could revert into a previous form of lower complexity. Such regresses are exceptional, at least in great steps, and are seen to some extent mainly amongst parasites. But maybe parasitism doesn’t necessarily lead to a decrease of complexity. As Conway Morris (2013 p. 150) suggests, the interlocking of the genomes of hosts and parasites seems to point towards an under-appreciated degree of complexity.

Another obstruction for a species to return to a previous level of lower complexity is understood in terms of genomic information content. A return to a lower level of complexity means a reduction of information, but “information cannot be lost /.../ because a mutation corrupting the information is purged due to the corrupted genome’s inferior fitness.” (Adami et al. 2000). In addition, it seems that in many cases when a species is confronted with severe environmental difficulties, it goes extinct instead of changing to a lower level of complexity.

Still another difficulty with Gould’s metaphor, is that it seems improbable that the drunkard after staggering to a certain distance from the wall, then suddenly should become sober enough to be able to follow a straight path parallel to the wall. Likewise, it seems to me improbable that a random diffusion process eventually should end up in the steady state situation that is observed in millions of species in millions of years. My main objection to an explication of increasing complexity by diffusion is that such a principle is incapable in explaining the well-known fact that most species don’t change much over their evolutionary history.

McShea is together with Robert Brandon (McShea and Brandon 2010) claiming that there is an even more fundamental biological law than natural selection: namely, the tendency for diversity and complexity to increase in evolutionary systems. They emphasize the central importance of increasing complexity by ranking it as biology’s first law, an idea that at a first look seems quite favorable to the present model. However, the authors claim that complexity may be increasing even without the action of natural selection. Their rationales and experimental tests in support of this contention are summarized by Zimmer (2013). Similarly, Kauffman (2013) attempts to explain complexity to emerge even without natural selection to help it along. In opposition to these views by Gould, McShea, and Kauffman, Lineweaver et al. (2013 p. 7) maintain that a definition of complexity as an increase in variation without the involvement of natural selection merely describes an increase in entropy, an approach to equilibrium rather than an increase of complexity.

## Complexity Versus Time

As we may conclude from the above extracts of the rich literature on complexity, a majority of scientists in the field maintain that evolution at large displays a trend towards increasing complexity. Edward O. Wilson makes these notions more explicit by observing that such a growth is observed to periodically rise upwards although in intermediate periods slow to a virtual halt. (Wilson 1992 p. 175). Furthermore, he points out that species emerge quickly and fully formed after rapid bursts of evolution, then persist almost unchanged for millions of years. (ibid. p. 80). Wilson bases his statements on initiated observations, and therefore I take his words as a basic empirical support for my theses.

In spite of the lacking measure and definition of complexity, I suggest Wilson’s anticipated features of increasing complexity may be illustrated by means of a diagram of complexity versus time as shown in Fig. 1. I have previously developed a similar diagram as a result of an analysis of the evolution of individual developmental courses (Ekstig 2010b). In the present approach, I give a complementary analysis of the diagram in terms of natural selection.

As a starting point, we may imagine a depiction of the evolutionary course of increasing and accelerating complexity by means of a curve, not unlike an exponential function (in the diagram shown as the stepwise curve). But there is a problem with such a general notion of growing complexity because, as is generally observed, far from all species follow such a trend. In fact, as stated by Wilson, a majority of species of all levels of complexity is by and large stable over time. Still more noticeable is that the oldest organisms of today, although being exposed to environmental contingencies and natural selection for the longest time, nonetheless are amongst the most primitive. These observations are, as we can see, consonant with the conundrum Lamarck expressed in the citations above. The intuitive idea of a generally growing complexity as driven by natural selection must therefore be revised. In the diagram, the more or less horizontal lines beneath the uppermost stepwise curve depict species that don’t change much.

These horizontal lines, representing the complexity of stagnant species, have their starting points connected to the stepwise curve. This feature illustrates the fact that organisms occurring first are the most primitive and that the more complex have appeared later. It also illustrates the thought that life on earth originated mainly once, because, as soon as the first forms were established, we may imagine that subsequent appearing forms were outcompeted. In evolution, as it is said, there are no hopeful monsters.

The stepwise curve represents the upper border of complexity and at the same time the common descent of all species, a central consequence of the present conception of the evolutionary process.

The diagram illustrates that major transitions occur at the steps in the curve representing the upper border of complexity. After such an elevation, the new species normally stabilizes at the new niche in the complexity space, thus forming a new horizontal line above those previously formed. This procedure is then repeated over and over again, leading to a cumulative elevation of complexity. At these steps, a portion of the ancestor population didn’t take part in the elevation, retaining its original level. Thus, when fish evolved into amphibians and reptiles, far from all fish species followed that enterprise. Most of them remained in the sea and in the diagram of Fig. 1, such occurrences are illustrated as a continuation of the straight lines from the lower level of each step.

Such a stepwise increase of complexity is by McShea (2001) analyzed in terms of levels of nestedness. In this conjecture, McShea maintains that stasis predominates with rare large steps upward at the maximum, a principle that, as we can see, agrees with the present analysis.

It must be emphasized, though, that the diagram is highly schematic, inasmuch as it attaches no scales to the axes and that the enormous number of species are represented by just a few lines, intended to indicate complexity of species at the reader’s choice. Furthermore, extinctions are not indicated, although it is easy to depict such occurrences by bringing corresponding lines to their ends at the point of time of extinction.

Another feature of the evolutionary process not displayed in the diagram is that there is a tremendous number of species deviating from the few lines shown in the diagram with their speciation occurring even after the separation of the highest borderline. Such deviations may be directed upwards as well as downwards but in each such occurrence, I think the changes in complexity are small in comparison to its increases to the highest borderline. This process has given rise to the rich diversity of life.

After this descriptive introduction of the diagram of Fig. 1, I proceed by discussing its explication.

## Natural Selection as the Motor of Increasing Complexity

I maintain that the natural variation of the functional capability of many features by natural selection is driven to successively higher levels of complexity. However, there is a problem with such a general notion of increasing complexity inasmuch as most species, as I already have indicated, after their emergence persist almost unchanged. The challenge is therefore to explain the significant increases of complexity in some lineages and the undeniable success of the great majority of species that do not change much over long times. I suggest that the solution of this problem lies in a closer interpretation of natural selection as acting on complexity. Fortunately, the necessary concepts for this analysis are already at hand.

**Fig. 2:**
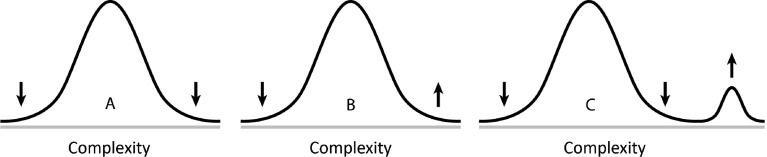
Three forms of natural selection. Curve *A* illustrates stabilizing selection, curve *B* directional selection, and curve *C* disruptive selection. What is new in this diagram is that the curves illustrate the spread of complexity. The *arrows* indicate how natural selection affects the form of the curves with time

It is common knowledge (see for instance Reece et al. 2011, p. 527) that natural selection works in three modes—stabilizing selection, directional selection, and disruptive selection—as illustrated in Fig. 2.

The traditional interpretation of these curves is that they illustrate the spread of phenotypic features. In the present context, the curves illustrate the spread of complexity, a view that gives the key to the interpretation of the diagram of Fig. 1. First, I discuss the application of *stabilizing selection*.

Stabilizing selection is what I think Dennett (1995 p. 89) refers to when he states that the odds are heavily against any mutation being more viable than the theme on which it is a variation. Actually, this idea was expressed already by Darwin (1859 p.121): “For it should be remembered that the competition will generally be most severe between those forms which are most nearly related to each other in habits, constitution, and structure.” Including complexity in the reasoning implies, I think, that a species, residing at a complexity level between the lowest and the highest levels (see Fig. 1), will meet high competition by species already established at adjacent levels, whether higher or lower, if it tries (so to say) to change its complexity level. Consequently, such a species is subject to a selection pressure compelling it to stay at the level of complexity it got at its emergence. This rationale explains the horizontal lines in the diagram of Fig. 1. In order to be able to display all the richness of animal species, each straight line the diagram of Fig. 1 should be divided into a great number of adjacent lines corresponding to nearly related species deviating upwards as well as downwards from the main line. In this way the development of parasites is included into the model as well. Such deviations are however small as compared to the steps in the uppermost stepwise line that drive evolution to significantly higher levels of complexity.

The next form of natural selection to be analyzed is *disruptive* *selection. *For a species thriving at the highest level of complexity at the actual point of time, there are no species at higher levels causing competition and therefore, if other conditions are favorable, nothing prevents it to evolve to such a higher level of complexity. But, there are certainly many traits that must harmonize in such a rise and therefore such a change occurs infrequently. Let us as an example return to the above discussion of the transition of the heart into four chambers. This transition must involve simultaneously occurring changes in anatomy, including not only changes of the heart’s different parts but changes in the rest of the body as well, each of which at all intermediate points of times not only should be functioning but also must bring about an advantage. No wonder that such transitions occur rarely.

Yet, when they happen, they may result in rapid bursts of evolutionary change, forming radically new species, depicted as steps upwards in the diagram of Fig. 1. After such an elevation, the species in most cases stabilizes at the new level, thus forming a new horizontal line above those previously formed. In this way, there will be a cumulative addition of species with successively higher levels of complexity forming the stepwise curve in the diagram of Fig. 1.

The third form of natural selection, *directional selection,* is characterized by a continuous change in a certain direction, as for instance in the universal trend of increasing body size. Such an increase of body size is by Bonner (1988) considered to be coupled to a growth of complexity but in this process, I think, the rise of complexity isn’t as great as that of disruptive selection as seen in the fact that in directional selection, the species retain their identities and names whereas in disruptive selection, new species with new names are generated. In the diagram of Fig. 1, this process should be expected to result in a minor slope of the horizontal lines, but since these changes are relatively small, I have omitted to indicate them in this highly schematic diagram.

I conclude that the three forms of natural selection explain rapidly increasing complexity as well as slowly changing and unchanging complexity.

These applications of natural selection may be associated to a discussion carried out by Conway Morris (2013 pp. 150–156), concluding that any room for exploring complexity much further is now highly restricted. Conway Morris presents several observations in support of the conclusion that there may be a limit to biological complexity and that these limits may be close to being reached. When applied to the present model, it means that the horizontal lines in the diagram lie very tight. One outstanding exception in Conway Morris’s view is the human species, which doesn’t seem to have reached the limits to its neural complexity and capacity.

As we have seen, I have in the above discussion of stabilizing selection suggested that many species by competition are compelled to keep to the level of complexity they once captured, and such a competition is of course harder if, as Conway Morris conjectures, the space of complexity is close to being filled. Furthermore, similarly to Conway Morris, I think that the human intelligence forms an exception to this way of reasoning inasmuch as it has opened a new landscape in the complexity space manifested by our cultural and scientific pursuits, hitherto showing no indication of saturation.

Another aspect of the discussed processes is conjectured by Paul Davies, implying that the growth of complexity is coupled to a directionality of branching. Davies suggests that if there is a deeper principle of complexification at work, then not only will the most complex representative of the biosphere tend to become even more complex over time, but the median of the complexity distribution will also shift towards higher values over time (Davies 2013 p. 32). The diagram in Fig. 1 can be seen as an illustration of this statement, whereas I think the envisaged deeper principle of complexification is nothing else but natural selection.

## The Tree of Life

As to the interpretation of the diagram of Fig. 1, the lines first and foremost are intended to indicate the levels of complexity of species, although they may as well be interpreted as visualizing the species per se, implying that the diagram can be seen as a Tree of Life. This metaphor, originating from Darwin himself, is widely used as a broad conception of the process of evolution. Its shape is widely discussed and many forms are suggested. But, as Dennett (1995 p. 88) asks, what would the overall shape of the entire Tree of Life look like, if we could take it all in at a glance? My answer is, as the reader certainly already has anticipated, that such shape is to be found in Fig. 1.

The foremost difference in comparison to prevalent forms is that the present form includes a specified dimension, complexity, perpendicular to the time dimension. Another difference is that my tree doesn’t show the appealing form of a more or less symmetric tree with the trunk corresponding to the common descent. Instead, common descent is in the present model found to be the lineage with the highest level of complexity, a lineage represented by the stepwise curve at the uppermost part of the diagram. In a certain meaning, all species can be said to be emanating from the very first forms of living organisms, since all have parts of their DNA conserved from these old organisms. I think that that is what Dawkins (1992 p. 263) has in mind when stating that all lineages have had exactly equal time to evolve since the dawn of life. However, in a macro-evolutionary sense, species identified by species-specific properties have consecutively emerged at specific points of time all along the evolutionary history, and this is what Dawkins specifies in detail in his seminal book *The Ancestor’s Tale* (Dawkins 2004).

In the book, Dawkins draws a lot of diagrams illustrating the evolutionary process on time scales, though suggesting no interpretation of the dimension perpendicular to the time scale. In the present analysis, I suggest this perpendicular dimension to be complexity. In Dawkins’s diagrams, the line of common descent as seen from the position of the human species is represented by a straight line labeled *already joined*. Following this line backwards, Dawkins identifies several meeting points, *rendezvous*, with lineages of other species. The line already joined corresponds in the diagram of Fig. 1 to the upper borderline and the rendezvous correspond to the emergence of new species at the steps in this line.

Each straight line in the diagram of Fig. 1 can be divided into a great number of adjacent lines corresponding to nearly related species identified in the consecutive diagrams in Dawkins’s book. I have refrained from depicting such subdivisions in my diagram because the number of species is so great that it is impossible to represent them in one and the same diagram.

## The Evolution of Language

The transition from biological to cultural evolution is commonly supposed to be instigated by the appearance of language.

Language is an exclusive feature of our species, qualifying us as the symbolic species (Deacon 1997). It can be regarded as a system of information-containing symbols, the interpretation and use of which require a high level of neural capability. The evolution of language has progressed gradually, stretching over several hundred thousand years. This however, is a short period of time as seen on the time scale of animal evolution and therefore, in the present context, I suggest this process can be regarded as an example of disruptive selection, bringing about a new step in the stepwise curve in the diagram of Fig. 1.

As to the mechanism behind the evolution of language, it is more or less tacitly understood that it has been driven by its great reproductive value, in other words, by natural selection. One may say that language made the brain visible for natural selection. The reproductive advantage of this process must have been great since it occurred in spite of the great nutritional cost for the building and maintenance of the big brain.

For the origin of language, Blackmore (1999 p. 106) suggests two principles: Imitation and sexual selection. She proposes that the crucial step in the origin of language was imitation, an obvious, if difficult to find, ‘good trick’, that is likely to arise in a species with good memory and a complex social life. Once found, imitation started a new kind of evolution in coevolution with the old. Then Blackmore suggests sexual selection to set in, because people will both preferentially copy and mate with people with the best language. In this way, the evolution of language was strongly enhanced.

Language is transferred from generation to generation by children’s learning from their parents. This means that the enhancement of language is driven by a forceful feedback process, leading to a self-reinforcing progress.

Another contribution, already mentioned, to the high rate of evolution of language can be found in the fact that information transition by memes is faster than that by matter as in the genetic process (Laszlo 2009). In addition, mankind’s culture certainly evolves much faster than somatic features of animals due of the open interactions between human societies in contrast to the reproductive isolation of animal species.

The evolution of language, paralleled by a never-ending growing ability of using symbols, brings us to the realm of human culture, comprising technology, agriculture, religion, art, and science—processes indicated by the line pointing unlimitedly upwards in the diagram. It could be observed that at the evolution of these cultural manifestations, the natural selection acting on genes has successively been replaced by the selection of memes. However, I will leave this topic for future discussions.

## The Human Species as a Unique Animal

The process of cumulative addition of species with successively higher levels of complexity forming the uppermost stepwise line in the diagram of Fig. 1 has a noteworthy implication. It implies that the latest emerging species on this line all along the evolutionary history will be the one with the highest level of complexity. At present, this species is we, human beings. New species have in fact emerged later then humans, as for instance the Bonobo (deviating from the Chimpanzee line) and certainly a lot of bacteria, but such deviations occur from lower levels of complexity and don’t contribute to the highest level of complexity.

The cumulative addition of species together with the evolution of language and culture gives us reason to place the human species at the highest position in the hierarchy of living creatures. This result concurs with the popular though intuitive notion of man as the summit of evolution. In fact, this contentious notion can be traced back to the medieval idea of the Great Chain of Being, indeed even to Aristotle. It is interesting to note that this obsolete idea comprises a hierarchical but not a time dimension, whereas many scientists of today, for instance Dawkins in *The Ancestor’s Tale* (Dawkins 2004), arrange life on a temporal but not on a hierarchical dimension. The present model attempts to use both these dimensions.

As I have posed above, the processes leading to the modern man have implied a much faster evolution than that of any other species, thus significantly broadening the border in the complexity space between us and all other species. Therefore, whereas the rest of living organisms, as I have discussed above, tightly fills the available complexity space, there is at present an empty space between us and other organisms, a gap previously filled by different kinds of hominids. But, being a really enigmatic fact, all these intermediate species are now extinct.

This empty gap, I think, makes it easier to embrace the notion of the uniqueness of the human species because one mustn’t draw the border in a continuum. This notion is however highly controversial, even fervently rejected, because, as has often been stressed, there is no objective basis on which to elevate one species above another. It is rather considered as an illusion founded on a chauvinistic expectation of human significance. Besides, as I have understood, there is a moral problem to it as well, because it seems to lead to the apprehension that if one admits the notion of man’s superiority over animals, one may perhaps be prepared to accept the idea that some ethnic groups are ahead of others as well. In the face of this highly contentious issue, I would like to refer to Diamond (1997) who seeks to explain Eurasian hegemony throughout history. He asserts that the gaps in power and technology between human societies do not reflect racial differences; rather they originate in initial environmental conditions.

I would like to strongly emphasize that the present model does not give reason for the conclusion that there be an objective basis on which to elevate on behalf of complexity one human race above another. Any society or race as a matter of fact consists of individual human beings employing life histories of highly different levels of complexity, more widely spread than are putative races. Therefore, I stress that a line representing the human species in the diagram of Fig. 1 cannot be divided into lines representing different societies or races on different levels of complexity. Thus, I find no reason to dismiss the view of mankind as a unique and superior species on the basis of fearing that such a view would allow for discrimination on ethnical grounds, a fear that seems to be part of the reason for the generally cautious attitude to the subject.

The view of mankind as a unique species is only rarely expressed in biological literature. However, Daniel Dennett in his broad survey over Darwinism boldly states:

People ache to believe that we humans are vastly different from all other species—and they are right! We are different. We are the only species that has an *extra* medium of design preservation and design communication: culture. That is an overstatement; other species have rudiments of culture as well, and their capacity to transmit information “behaviorally” in addition to genetically is itself an important biological phenomenon, /.../ but these other species have not developed culture to the takeoff point the way our species has. (Dennett 1995 p. 338)

Furthermore he states that cultural evolution operates many orders of magnitude faster than genetic evolution, and this is part of its role in making our species special. (ibid p. 339)

Likewise, American philosopher George Kateb, in his ambition to strengthen human dignity, ardently expresses the superiority of mankind amongst all species:

We human beings belong to a species that is what no other species is; it is the highest species on earth—so far. /.../ All other species are more alike than humanity is like any of them; a chimpanzee is more like an earthworm than a human being, despite the close biological relation of chimpanzees to human beings. The small genetic difference between humanity and its closest relatives is actually a difference in capacity and potentiality that is indefinitely large, which actually means that it can never be fully measured. (Kateb 2011 p. 17)

## Summary

I have in this thesis suggested a rough and qualitative description of complexity in biology coupled to the level of functional capability of the inner organs, the nervous system and intelligence. I have furthermore suggested that increasing complexity is explained by natural selection and I have shown that these rationales have brought about explications of some contentious problems involved in the basic understanding of the evolutionary process.

First and foremost, the reasoning has led to a solution of a difficulty in the common notion of a generally increasing complexity, namely the annoying observation that most species haven’t changed much since they came into existence. This state of affairs is explained as a consequence of competition by already established species on adjacent levels of complexity. At the same time, natural selection has raised a limited number of species residing on the highest level of complexity to still higher levels of complexity, because for these, there are no competing species at higher levels. In this way the present analysis is in better concord with observations than the widespread notion of a general and unspecific growth of complexity.

The suggested model makes possible the construction of a new form of the Tree of Life, visualized by a highly schematic diagram of complexity versus time (Fig. 1), qualitatively displaying the levels of complexity over time in living creatures including the human species. In this diagram, the generally observed trend towards higher complexity is illustrated by the step-formed curve representing the upper limit of complexity. Beneath this limit, the complexity space is filled with an overwhelming number of stagnant species. However, there is an exception to this situation inasmuch as there is an empty gap in complexity between mankind and all other species, previously filled with now extinct hominids.

Furthermore, the model implies that the steps to higher levels of complexity follow each other in a cumulative way, rendering evolution a direction in time, an arrow of time. This means that evolution began with species at the lowest level of complexity, followed by species of successively higher levels of complexity. This may seem intuitively self-evident but many scientists nevertheless hesitate about how to express it. In addition to the explanation of the direction of evolution, the suggested model also gives a view of the diversity of life as coupled to natural selection and complexity.

Another result of the present model is that the common descent of all organisms is manifested by the lineage with the highest level of complexity throughout the history of evolution, a lineage represented by the stepwise curve in the diagram of Fig. 1.

Finally, a noteworthy inference of the model is that the cumulative addition of complexity implies that the latest formed species is the one with the highest complexity. At present, this species is we, the human species. This conclusion is by biologists seen as highly controversial, mainly I think, due to the lacking definition of the concept of complexity. But at least, I venture to say, it isn’t to say too much to state that we have started a new epoch in the evolution of life on earth, characterized by the emergence of advanced language, technology, religion, science, and art; faculties no other species has come close to. The present model thus implies that we constitute an integral part of organic evolution, yet rendering us the exclusive status as the species of the highest complexity.
